# Construction and Characterization of an Intergeneric Fusant That Degrades the Fungicides Chlorothalonil and Carbendazim

**DOI:** 10.3389/fmicb.2022.842736

**Published:** 2022-03-10

**Authors:** Chen Xue, Jiaxin Zheng, Guangli Wang, Liang Feng, Feng Li

**Affiliations:** College of Life Sciences, Huaibei Normal University, Huaibei, China

**Keywords:** chlorothalonil, carbendazim, RAPD, protoplast fusion, degradation

## Abstract

*Bordetella* sp. CTN-16 (GenBank FJ598326) can degrade chlorothalonil (CTN) but not carbendazim (MBC), and *Microbacterium* sp. MBC-3 (GenBank OK667229) can degrade MBC but not CTN. A functional strain BD2 was obtained by protoplast fusion of CTN-16 and MBC-3 to generate a fusant with improved degradation efficiency of CTN and MBC. Fusant-BD2 with eighth transfer on a medium containing CTN and two antibiotics was obtained. To identify and confirm the genetic relationship between parental strains and fusion strain BD2, scanning electron microscopy (SEM), random amplified polymorphic DNA (RAPD), and 16S ribosomal RNA (rRNA) gene sequences analysis were carried out. SEM analysis illustrated BD2 and its parents had some slight differences in the cell morphology. Fusant-BD2 not only possessed the same bands as parental strains but also had its specific bands analyzed through RAPD. The genetic similarity indices for BD2 and its parental strains CTN-16 and MBC-3 are 0.571 and 0.428, respectively. The degradation rates of CTN and MBC were 79.8% and 65.2% in the inorganic salt solution containing 50 mg·L^−1^ CTN and 50 mg·L^−1^ MBC, respectively, and the degradation efficiencies were better than the parental strains CTN-16 and MBC-3. This study provides a prospect for the application of fusion strain BD2 in bioremediation of CTN and MBC contaminated sites.

## Introduction

Chlorothalonil [2, 4, 5, 6-tetrachloroisophthalonitrile (CTN)] is a non-systemic broad-spectrum fungicide that has been used to prevent and control various crop fungal diseases (Tang et al., 2017). In China, CTN is often used in greenhouses for various vegetables, fruits, rice, and other cash crops or to prevent lawn fungal foliar disease (Sakkas et al., 2002; Wu et al., 2012). CTN is highly toxic to birds and aquatic invertebrates (Ren et al., 2011). It has been reported to cause serious eye and skin irritation, harm human gastrointestinal tract, and proved to be carcinogenic to human (Wang et al., 2011). CTN is easily absorbed by the soil and it has a half-life of up to 100 days, which could cause great pollution to soil and environment (Shi et al., 2010). Therefore, increasing concerns about the toxicity of CTN has resulted in the demand for novel methods to remove environmental CTN contamination safely and effectively. Microbial degradation is the most efficient method for metabolizing CTN in the environment (Liang et al., 2010). Several microorganisms have been reported to co-metabolize low concentrations of CTN in the presence of other nutrient substances (Chaves et al., 2007; Liang et al., 2011). However, some research groups have isolated strains of CTN-degrading bacteria showing the capacity to degrade CTN without other substrates (Liang et al., 2010; Yue et al., 2015).

Carbendazim [methyl-1H-benzimidazol-2-ylcarbamate (MBC)] is a systematic broad-spectrum fungicide with stable chemical properties (Sun et al., 2014). It is highly used in China partly due to its highly stable chemical properties (Xiao et al., 2013). It has been reported that MBC residues and accumulation in fruits, vegetables, crops, and soil can affect human health through the food chain (Ji et al., 2016). MBC is very stable in soil and water with a relatively slow degradation rate (a half-life of up to 12 months). Excessive use of MBC could change the structure of soil microbial community and result in soil pollution (Cuppen et al., 2000). Therefore, it is important to construct fused strains that efficiently degrade MBC. Microbial degradation has been an effective strategy. Until now, several microorganisms have been isolated, such as *Rhodococcus* sp. D-1 (Bai et al., 2017), *Rhodococcus* sp. CX-1 (Long et al., 2021). *Microbacterium* sp. djl-6F (Lei et al., 2017), *Mycobacterium* sp. strain djl-10 (Zhang et al., 2017a), *Pseudomonas* sp. CBW (Fang et al., 2010), *Rhodococcus jialingiae* djl-6-2 (Wang et al., 2010b).

However, due to the existence of a variety of fungicide residues in the soil environment, single microbial degradation is not enough due to low degradation efficiency and narrow degradation spectrum (Wang et al., 2016). Therefore, it is extremely beneficial to construct a fusion strain that can degrade both CTN and MBC. Interspecific protoplast fusion is a useful strategy to construct an improved efficiency fusant (El-Gendy et al., 2017). This strategy can effectively carry out specific bioremediation of common fungicides in polluted environment. Moreover, the multifunctional degradation bacteria can compensate for the degradation of single pesticide species in the environment by domestication and selection of indigenous microorganisms.

Protoplast fusion is one of the methods of microbial breeding. It can transfer genes from one strain to another, creating genetically engineered strains. The DNA of the parent strain can be fused with the protoplast to obtain a new fusion. Advantages of using protoplast fusion include small breeding restriction, complete transfer of directional genetic material, and good directional property. It has been widely and effectively applied in the research of biological treatment of industrial pollutants. Chen et al. (2011) used protoplast fusion technology to construct a new strain that could effectively degrade chlorophenol (Chen et al., 2011). Feng et al. (2013) constructed a functional strain F1, which simultaneously enhanced its ability to degrade methyl benzensulfuron and butachalamine by using protoplast fusion technology (Feng et al., 2013). On the other hand, Wang et al. (2016) constructed a functional strain AC, which improved its ability to degrade acetaminprid and chlorothalonil simultaneously to a certain extent through protoplast fusion (Wang et al., 2016). However, to the best of our knowledge, no bacterial species have been reported that degrade both CTN and MBC.

This study aimed to cultivate a novel fusion bacterium using interspecific protoplast fusion capable of degrading CTN and MBC and to characterize its morphological, physiological, and biochemical characteristics. Genetic relationships between protoplast fusion strains and their parents were analyzed using random amplification polymorphic DNA (RAPD). Subsequently, the degradation ability of the fused bacteria was also studied.

## Materials and Methods

### Parental Strains

The natural bacterium MBC-3 (GenBank OK667229) was screened from soil samples of a vegetable greenhouse in Huaibei District, Anhui Province, by classical enrichment techniques and identified based on Bergey’s Manual of Determinative Bacteriology and 16S rRNA gene sequence analysis (Buldyrev et al., 1995; Che et al., 2009). The strain of CTN-16 (GenBank FJ598326) used in the study had been formerly obtained from CTN polluted soils in our research group (Liang et al., 2011).

### Chemical Reagents and Media

CTN (>99% purity) and MBC (>98% purity) were purchased from J & K scientific Ltd. (Shanghai, China). Lysozyme was purchased from Sangon Biotech Co., Ltd. (Shanghai, China). Reagents and solvents used in this study are all analytical grades. The methods employed on the use of inorganic salt medium and RNB regeneration medium are according to previous literature reports (Feng et al., 2013; Hu et al., 2021).

### Protoplast Fusion

The sloped-activated bacteria were cultured in a liquid bacterial medium until the logarithmic growth stage and then 0.5% glycine was added until the OD_600nm_ = 1.5. Lysozyme (1 mg·ml^−1^) was added to the shaker and cultured overnight. SMM (sulfamethoxine sodium in methanol) stabilized solution (Wang et al., 2016) was added the next day to form protoplast suspension. These experiments were handled gently to avoid damage to the protoplasts. Then added were 0.5 ml newborn calcium phosphate solution and a preheated (35°C) 40% PEG 4000 solution. The solution was held for 10 min in a 30°C water bath. Finally, the bacterial solution was diluted and coated on RNB medium including 50 mg·L^−1^ kanamycin and ampicillin, and 200 mg·L^−1^ CTN. The plates were cultured at 30°C for 24 h and stored at 20°C for 48 h.

### Isolation and Identification of Fusion Bacteria

The fusants were screened using a selective medium (the RNB medium containing 50 mg·L^−1^ kanamycin and ampicillin, and 200 mg·L^−1^ CTN) according to Wang et al. (2010a), that is, colonies with transparent halos were scored positive (Wang et al., 2010a). To confirm the MBC degradation ability of the strain, the objective strains were used to inoculate into the inorganic salt solutions containing both 100 mg·L^−1^ MBC and CTN. The fusants capable of degrading MBC and CTN were the desired degradation strains.

To test the genetic growth stability of the fusion bacteria, the screened fusion bacteria were inoculated into the RNB regeneration medium containing antibiotics (Kanamycin and Ampicillin) and bactericides (CTN and MBC) and subcultured at 30°C more than eight times. Subsequently, the morphological characteristics of the fused bacteria, including colony morphology were determined by the streaking plate method, and the structure morphology was analyzed by scanning electron microscope (SEM). Other physiological and biochemical characteristics of the fused strains were determined according to the method described by Wang et al. (2010a).

Total DNA was extracted and purified by Sangon Biotech Co., Ltd. (Shanghai, China). The primer pairs 27F/1492R were used to quantify bacterial 16S rRNA gene sequences in all samples, respectively (Long et al., 2021). Amplification was done under the following conditions: 94°C for 5 min, 94°C for 30 s, 55°C for 30 s, 72°C for 90 s, and final extension at 72°C for 10 min. The PCR products were purified and sequenced at General Biotechnology Co., Ltd. (Chuzhou, China). The neighborhood connection method of MEGA X software was used to construct a phylogenetic tree (Kumar et al., 2018; Sudhir et al., 2018).

### Random Amplified Polymorphic DNA PCR

Forty random primers used in this study were presented in Table 1. The optimized RAPD-PCR reaction system was carried out in 20 μl volume, containing 10 μl Taq PCR Master Mix (2X, blue dye) including Taq DNA polymerase, dNTP, MgCl_2_, PCR buffer, PCR stabilizers, gel loader and bromophenol blue, 1 μl random primer and 1 μl DNA template of the strains, and purified water to the final volume. RAPD-PCR amplification was implemented under the following conditions: 94°C for 5 min, 35 cycles at 94°C for 30 s, 31°C for 45 s, 72°C for 90 s, and final extension at 72°C for 10 min, then repeat the above steps 35 times. PCR products (5 μl) and DNA markers were analyzed by 1% agarose gel electrophoresis. The gel was run at 120 V for 0.5 h. The gels were photographed using UV in the Tanon-1,600 digital gel image analysis system (Shanghai Tianeng Technology Co., LTD, Shanghai, China).

**Table 1:** Sequences of arbitrary primers.

Number	Sequence	Number	Sequence
1	TGCGCCCTTC	21	TGGCCCTCAC
2	GTCCACACGG	22	CTGGCGAACT
3	CCACAGCAGT	23	GTCCGGAGTG
4	CAGGCCCTTC	24	CCCAGCTGTG
5	GGTCCCTGAC	25	CATACCGTGG
6	CTCACCGTCC	26	ACCGCCTGCT
7	AGGGCCGTCT	27	CACCATCCGT
8	TGCCCGTCGT	28	CCCAGTCACT
9	CCCGATTCGG	29	GGCATGACCT
10	CCGCCTAGTC	30	ACAGCCTGCT
11	TGACGGCGGT	31	AGGCTGTGCT
12	TCTCCGCCCT	32	CAGTGCCGGT
13	TGCCCAGCCT	33	CTGGGTGAGT
14	GGCAGGCTGT	34	GTGACAGGCT
15	GGACGGCGTT	35	GTCCACTGTG
16	TGGGTCCCTC	36	CCTCCAGTGT
17	ACGGGCCAGT	37	CACCCCAGTC
18	GGGTCTCGGT	38	TCCCATGCTG
19	GAGCGAGGCT	39	CTGGGCACGA
20	GTCCCGTGGT	40	TGAGGGTCCC

### Gel Statistical Analysis

According to the electrophoretic band type of RAPD products, bands with the same migration distance were regarded as the same site, and those with the occurrence of bands were denoted as 1, and those without were denoted as 0. The bands of fusion bacteria BD2, CTN-16, and MBC-3 were counted to construct the “0–1” binary data matrix. NTsys-2.0 software for clustering was used to automatically generate genetic clustering tree (Nei and Li, 1979; Feng et al., 2013).

### Biodegradation Experiments

CTN-16, MBC-3, and fused bacteria were placed in LB liquid medium for culture activation. To determine the optimal conditions for rapid growth and biodegradation of BD2, for temperature conditions: BD2 was inoculated in inorganic salt solution at culture temperature (26–34°C, 2°C unit increase) and its growth was observed. For the determination of pH value, BD2 was inoculated into inorganic salt solution with different pH values (4.5–10.5, with an increase of 1.0 unit) to observe its growth status. After the cell density had been adjusted to about 1.0 × 10^8^ CFU·ml^−1^, the aliquot BD2 cells of 1%, 3%, 5%, and 7% were inoculated into inorganic salt solution, respectively in order to study the effect of the initial inoculum size on the biodegradation of BD2.

The cultured bacterial solution was inoculated in the same amount into the inorganic salt medium containing CTN and MBC with concentrations of 50, 100, and 200 mg·L^−1^. All treatments of both the control and experimental groups were conducted in triplicates. The control groups were only added with fungicides as the control standard, while the experimental groups were added with bacterial solution and fungicides of the same concentration. All the experimental solutions were placed in 30°C and 160 rpm shaker for 5 days. After culturing, CTN and MBC samples were prepared and analyzed following Wang et al. (2010a).

The following experiments were performed to analyze the degradation of other fungicides by parent bacteria and fusion sons. Taking diniconazole as an example, two replicates were set for both the control group and the experimental group. The control group was only added with diniconazole as the control standard, and the experimental groups were added with the same concentration and volume of parent bacteria solution and fusant solution. Then, equal amounts of diniconazole were added. All experimental solutions were incubated in a 30°C, 160 RPM shaker for 7 days. After culturing, samples were prepared according to the method of Zhang et al. (2018) and degradation was analyzed by UV detection or HPLC (Zhang et al., 2018). The experimental methods of other fungicides (propiconazole, hexazolol, tebutazol, etc.) were similar to the above methods (data not shown).

## Results and Discussion

### Isolation and Identification of Native Bacterium MBC-3

**Figure 1:**
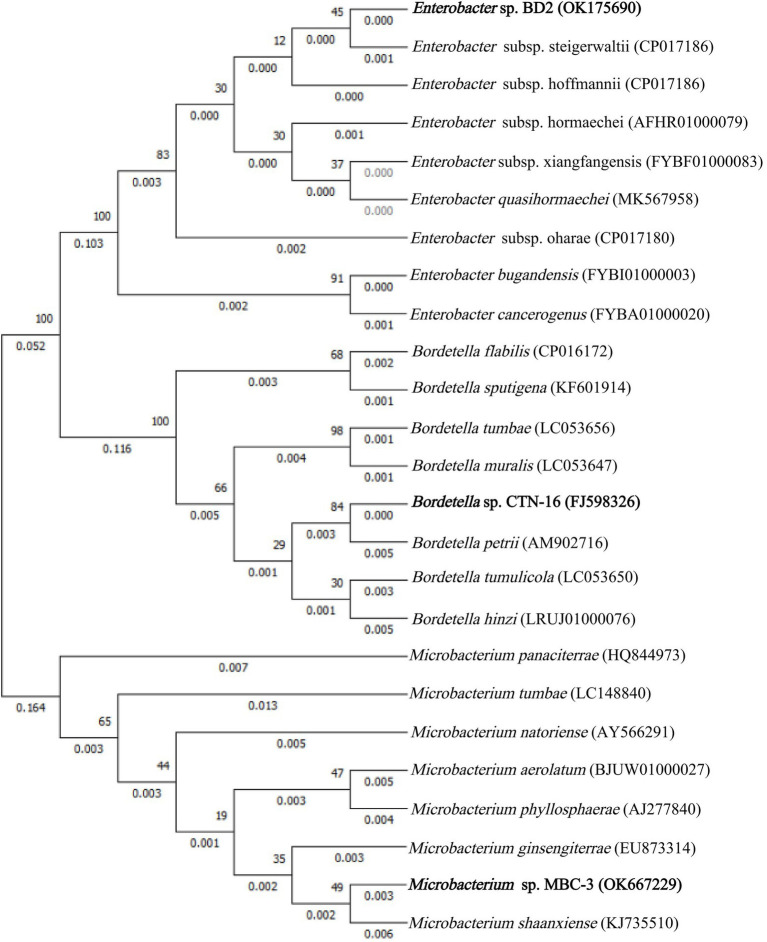
Phylogenetic dendrogram of 16S rDNA sequence constructed using MEGA X software.

In this experiment, with MBC as the only carbon and energy source, several strains of MBC degrading bacteria were isolated, and the strain MBC-3 with high degradation efficiency was selected for further study. Phylogenetic analysis of 16S rRNA gene sequence showed that strain MBC-3 belonged to *Microbacterium* species and formed a subclade with *Microbacterium Shaanxiense* KJ735510^T^ (similarity = 99.8%) with a high bootstrap value of 95.9% (Figure 1). Thus, strain MBC-3 was identified as a *Microbacterium* sp. Previous studies have indicated that it can degrade MBC. In addition to this, the isolate, *Microbacterium keratanolyticum* ZY, was expressed for Dichloromethane (DCM) degradation. *Microbacterium* sp. C448 can be used to degrade sulfamethoazine. *Microbacterium* esteraromaticum SBS1-7 can biodegrade toluene/styrene. *Microbacterium* sp. WHC1 can effectively remove Ciprofloxacin (CIP) for biological repair (Billet et al., 2021; Hu et al., 2021; Sodhi et al., 2021; Wongbunmak et al., 2021).

### Screening and Identification of Bacterial Fusant

Rudimentary antibiotic growth/inhibition experiments showed that parent CTN-16 was sensitive to kanamycin and resistant to ampicillin, contrary to the antibiotic properties of parent MBC-3. The fusion bacteria could grow on both kanamycin and ampicillin at the same time, indicating it inherited the antibiotic properties of the two parent strains. A transparent circle was used as a marker of CTN degradation to screen a fusion promoter for CTN degradation. Stability test results showed that some colonies disappeared after 4–5 rounds of transfer, while others still maintained the ability to grow on selective plates after eight rounds of transfer. A stable putative fusion strain that grew after eight rounds of transfer was identified and designated as fusant-BD2, which produced a distinct transparent circle on a selective agar plate containing 200 mg·L^−1^ CTN.

**Figure 2:**
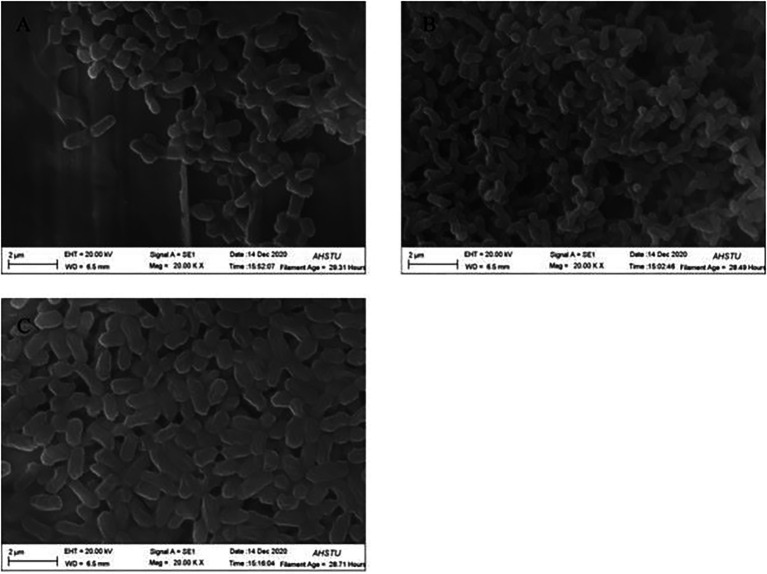
Scanning electron microscope (SEM) morphology of bacterial hybrid **(A)** CTN-16, **(B)** MBC-3, and **(C)** BD2.

Scanning electron microscopy (SEM) has been extensively used in biological experiments and has become an effective way to confirm protoplast fusion (Zhao et al., 2009; Sun et al., 2013). As shown in Figure 2, the cell morphology of BD2 is somewhat different from that of the two parents. The parent strain CTN-16 was bulbous whereas MBC-3 is elongated. The fused bacterium BD2 was straight rod-shaped.

The similarities and differences between fusion bacterium BD2 and its parents are presented in Table 2. The comparison of morphological, physiological, and biochemical characteristics showed that the fusion bacterium BD2 manifested some genetic characters of its parents. It was confirmed that the fusion strain BD2 contained the genomic DNA of the parental strains, and PCR amplification and automatic DNA sequencing were performed on 16S rDNA at General Biotechnology Co., Ltd. (Chuzhou, China). We found the known closest relationship with BD2 searching in GenBank and constructed a phylogenetic tree based on the 16S rDNA of the fusion strain BD2 and the parents. The comparison and identification of microbial species were carried out on BANKIT. Comparing its 16S rDNA, the fusion strain BD2 was identified as *Enterobacter* sp. The 16S rDNA of CTN-16 has been listed in GenBank with the accession number FJ598326 and the 16S rDNA of MBC-3 was listed as OK667229 in GenBank, and the fusion order of 16S rDNA of fusion bacterium BD2 has also been listed in the GenBank with the GenBank ID OK175690.

**Table 2:** Morphologic and physio-biochemical differences between fusant BD2 and its parents.

Characteristic	BD2	CTN-16	MBC-3
Morphology	Yellow, wet	Yellow, wet	Yellowish, wet
Growth temperature (°C)	15–42	15–40	10–40
NaCl range for growth (%, w/v)	0–10	0–6	0–6
pH range for growth	4–12	5–9	3–12
Glucose	P	P	P
Maltose	P	P	P
Lactose	P	P	P
Inositol	P	P	N
Motility	P	P	N
Gram strain	N	N	P
Indole production	P	P	N
Kanamycin	S	S	R
Kmpicillin	R	R	S
Geneticin	S	S	R
MBC degradation	P	N	P
CTN degradation	P	P	N
DNA G + C content (mol %)	56.8	63.7	67.1

*P, positive; N, negative; R, resistant; S, sensitive.*

Previously, it was reported that *Enterobacter* strains can degrade some environmental pollutants. Zhan et al. (2020) isolated a TMX-6 strain capable of degrading thiamethoxam (TMX) and identified it as *Enterobacter cloacae* (Zhan et al., 2020). Thangaraj et al. (2021) isolated a strain of *Enterobacter hormaechei* SKB16 that can degrade azo dyes effectively (Thangaraj et al., 2021). Ekram et al. (2020) isolated *Enterobacter* sp. from paddy soil and it was confirmed that the isolated bacteria had the ability to degrade carbofuran (Ekram et al., 2020). Sun et al. (2019) isolated a rod-shaped bacterium *Enterobacter* sp. DNB-S2 that can efficiently degrade di-n-butyl phthalate (DBP; Sun et al., 2019). Chávez et al. (2016) found that *Enterobacter* isolated from stool samples of infants can efficiently degrade intestinal bacteria (Chávez et al., 2016). This is the first report of an *enterobacter* sp. that can decompose CTN and MBC simultaneously.

### RAPD Fingerprint Analysis

**Figure 3:**
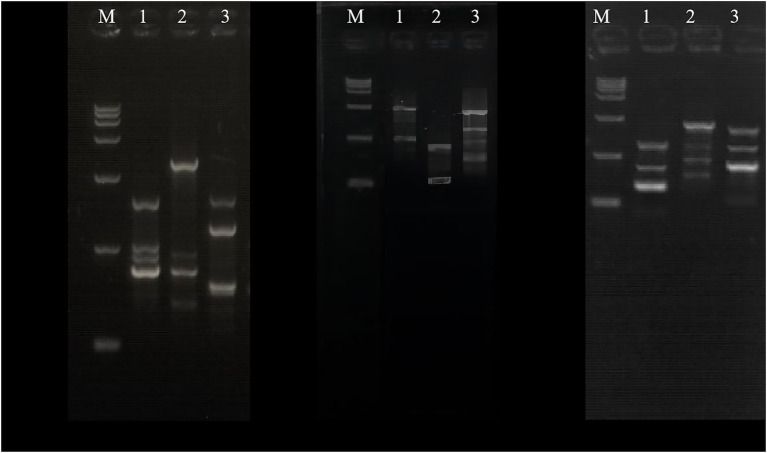
Electrophoresis of primers 11, 12 and 18, Lane1, CTN-16; lane 2, MBC-3; and lane 3, BD2.

In existing studies, PCR-RAPD analysis has been proved to be a dependable and easy method to calculate the genetic distance between the parents and the fusion (Savitha et al., 2010; Enan, 2011; Maharani and Dyah, 2012; Drożdż et al., 2015; Oluborode et al., 2018). The genomic DNA of the parents and the fusant-BD2 were amplified by PCR, and the results are shown in Figure 3. The lengths of the amplified fragments of the genomic DNA of the parents CTN-16 and MBC-3 and their fusant-BD2 of each primer were between 100 and 2,000 bp, and the amplified RAPD band spectra were significantly different. There are also some differences in the amplified bands of the two parent strains and their fusion using the same primer. The amplified strips of fusion BD2 are the same as CTN-16 and some stripes are also the same as MBC-3, indicating that the genomic DNA of fusion BD2 has sequences homologous to the genome of CTN-16 and MBC-3. It was observed that the CTN-16 and MBC-3 were recombined at the genome level, and the fusion strain BD2 was obtained. This indicates there are indeed genomic differences between the fusion strain BD2 and the parental strains.

**Figure 4:**
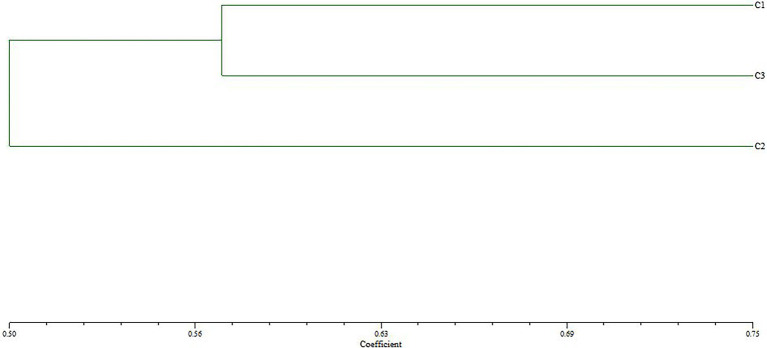
Cluster tree analysis of fusion fungus BD2 and its parents CTN-16 and MBC-3 constructed using UPGMA method (C1, CTN-16; C2, MBC-3; and C3, BD2). The scale value at the bottom is the UPGMA coefficient, which represents the percentage of similarity between BD2 and its parents.

The genetic relationship between BD2 and its parental strains CTN and MBC was analyzed by the RAPD method (Savitha et al., 2010; Tan et al., 2010). Primers 05, 07, 11, 18, 29, 33, and 40 were selected in the experiment. One hundred thirty-three clear and consistent bands were obtained through analysis. The results showed that there were both common bands and characteristic bands between the fusion bacteria and the parents, indicating that the genetic basis of each strain was roughly the same and the existence of genetic differentiation, respectively (Zhao et al., 2009). The genetic similarity index between BD2 and its two parents CTN and MBC was calculated by NTsys-PC software, and the tree graph of genetic relationship was established by the unweighted paired arithmetic average (UPGMA) method. The genetic similarity indexes between CTN-16 and fusion strain BD2 and MBC-3 and fusion strain BD2 were 0.571 and 0.428, respectively, indicating fusion strain BD2 was more inclined to CTN-16 in terms of genetic inheritance (Figure 4).

### Degradation Ability

**Figure 5:**
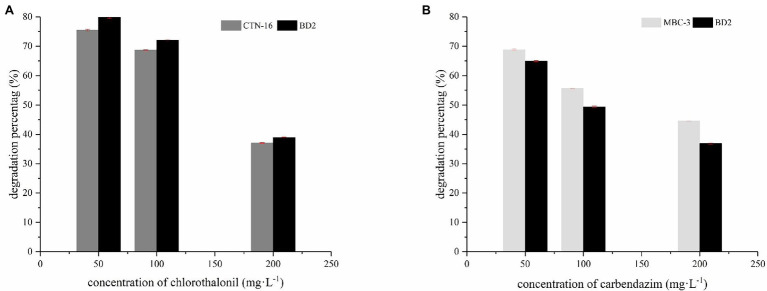
Degradation of two pesticides: the degradation percentage of CTN-16 and BD2 to CTN **(A)**; the degradation percentage of MBC by MBC-3 and BD2 **(B)**.

In our preliminary experiments, we found out that the optimum growth conditions and the maximum degradation for BD2 are a temperature of 30°C, pH of 7.5, and inoculation amount of 5% (data not shown). As shown in Figure 5, CTN and MBC are degraded by fusion bacterium BD2 and its parent at pesticide substrate concentrations of 50, 100, and 200 mg·L^−1^. After 5 days, the degradation rate of BD2 to CTN was 79.2% and that of MBC was 64.5% at the concentration of 50 mg·L^−1^ substrate. At the substrate concentration of 100 mg·L^−1^, the degradation rate of BD2 to CTN was 72.6%, while the degradation rate of BD2 to MBC was 49.9%. And, a degradation rate of 38.5% for CTN and 36.4% for MBC was recorded when the substrate concentration was 200 mg·L^−1^. In addition, compared with the two mothers, the fusion had a stronger ability to degrade CTN, but a weaker ability to degrade MBC. Therefore, BD2 showed good and stable degradation efficiency for the degradation of CTN and MBC, which is of great significance. However, the degradation properties of different strains to CTN and MBC are inconsistent. For example, *Mycobacterium* sp. SD-4 can degrade 50 mg·L^−1^ MBC with an average degradation rate of 0.63 mg·L^−1^ h^−1^ while *Pseudomonas* sp. CBW can degrade 0.14 mg·L^−1^ h^−1^ (Zhang et al., 2017b). *Stenotrophomonas* sp. H4 removed 82.2% of CTN in 7 days with an average degradation rate of 0.49 mg·L^−1^ h^−1^ (Zhang et al., 2014). The average degradation rate of *Pseudomonas* sp. AC was 2.52 mg·L^−1^ h^−1^ (Feng et al., 2013). In this study, the degradation rate of CTN-16 and MBC-3 was 0.56 mg·L^−1^ h^−1^ and 0.46 mg·L^−1^ h^−1^, respectively, while the average degradation rate of BD2 to CTN was 0.61 mg·L^−1^ h^−1^ and that of BD2 to MBC was 0.43 mg·L^−1^ h^−1^, indicating the fusion bacteria could degrade both CTN and MBC simultaneously.

After degradation experiments, data analysis shows that the two parent bacteria could only degrade their own corresponding fungicides, but could not degrade other fungicides such as diniconazole, tebuconazole, propiconazole, hexaconazole, and so on. The fusant BD2 can only degrade CTN and MBC, but the fusant BD2 cannot degrade other fungicides mentioned above (data not shown).

## Conclusion

The strain fusant BD2 constructed by intergeneric fusion between *Bordetella* sp. CTN-16 and *Microbacterium* sp. MBC-3 was isolated and identified by antibiotic test, SEM, and RAPD. It was confirmed that MBC-3 had good degradation ability to CTN and MBC. This study pointed out the potential role of fusion bacteria in the development of new bioremediation strategies in the co-polluted environment of CTN and MBC, indicating the hybrid bacteria has a clear and broad application prospect in the bioremediation of a variety of contaminated soil. Of course, whether mixed bacteria can produce good effects in a complex field environment is still uncertain, and further research is needed.

## Data Availability Statement

The datasets presented in this study can be found in online repositories. The names of the repository/repositories and accession number(s) can be found in the article/supplementary material.

## Author Contributions

CX conducted the experiments, analyzed the data, and wrote the manuscript. JZ conducted the experiments. GW designed the study and wrote the manuscript. LF analyzed the data. FL analyzed the data and revised the manuscript. All authors contributed to the article and approved the submitted version.

## Funding

This project received a support from Provincial Natural Science Foundation of Anhui (2108085MC70) and the Natural Science Foundation from the Educational Commission of Anhui Province (KJ2020B04).

## Conflict of Interest

The authors declare that the research was conducted in the absence of any commercial or financial relationships that could be construed as a potential conflict of interest.

## Publisher’s Note

All claims expressed in this article are solely those of the authors and do not necessarily represent those of their affiliated organizations, or those of the publisher, the editors and the reviewers. Any product that may be evaluated in this article, or claim that may be made by its manufacturer, is not guaranteed or endorsed by the publisher.
